# A compound downregulation of *SRRM2* and miR-27a-3p with upregulation of miR-27b-3p in PBMCs of Parkinson’s patients is associated with the early stage onset of disease

**DOI:** 10.1371/journal.pone.0240855

**Published:** 2020-11-10

**Authors:** Soudabeh Fazeli, Majid Motovali-Bashi, Maryam Peymani, Motahare-Sadat Hashemi, Masoud Etemadifar, Mohammad Hossein Nasr-Esfahani, Kamran Ghaedi

**Affiliations:** 1 Department of Cell and Molecular Biology and Microbiology, Faculty of Biological Science and Technology, University of Isfahan, Isfahan, Iran; 2 Department of Biology, Faculty of Basic Sciences, Islamic Azad University, Shahrekord, Iran; 3 Department of Animal Biotechnology, Cell Science Research Center, Royan Institute for Biotechnology, ACECR, Isfahan, Iran; 4 Department of Neurology and Isfahan Neurosurgery Research Center, School of Medicine, Isfahan University of Medical Sciences, Isfahan, Iran; Hokkaido Daigaku, JAPAN

## Abstract

Parkinson’s disease (PD) is diagnosed when motor symptoms emerges, which almost 70% of dopamine neurons are lost. Therefore, early diagnosis of PD is crucial to prevent the progress of disease. Blood-based biomarkers, which are minimally invasive, potentially used for diagnosis of PD, including miRNAs. The aim of this study was to assess whether SRRM2 and miR-27a/b-3p could act as early diagnostic biomarkers for PD. Total RNAs from PBMCs of 30 PD’s patients and 14 healthy age and gender matched subjects was extracted. The expression levels of respective genes were assessed. Data were presented applying a two-tailed unpaired *t*-test and one-way ANOVA. We observed significant down-regulation of *SRRM2* (*p* = 0.0002) and miR-27a-3p (*p* = 0.0001), and up-regulation of miR-27b-3p (*p* = 0.02) in PBMCs of Parkinson's patients. Down-regulation of miR-27a-3p is associated with increasing disease severity, whereas the up-regulation of miR-27b-3p was observed mostly at HY-1 and disease duration between 3–5 years. There was a negative correlation between SRRM2 and miR-27b-3p expressions, and miR-27a-3p positively was correlated with miR-27b-3p. Based on functional enrichment analysis, *SRRM2* and miR-27a/b-3p acted on common functional pathways. miR-27a/b-3p could potentially predict the progression and severity of PD. Although both miRs had no similarity on expression, a positive correlation between both miRs was identified, supporting their potential role as biomarkers in clinical PD stages. Of note that SRRM2 and miR-27a-3p were able to distinguish PD patients from healthy individuals. Functional analysis of the similarity between genes associated with SRRM2 and miR-27a/b-3p indicates common functional pathways and their dysfunction correlates with molecular etiopathology mechanisms of PD onset.

## Introduction

Parkinson’s disease (PD) is defined as a chronic and progressive neurodegenerative disorder with destruction of dopaminergic neurons in the *substantia nigra* from ventral midbrain mainly by accumulation of Lewy body inclusions [1, 2]. Clinical manifestations of PD are bradykinesia, resting tremor, rigidity, and failure of postural reflexes [3]. Before the appearance of motor symptoms, non-motor manifestations are prognostic signs of disease which prove the systemic nature of PD [4]. In this step, early diagnosis of PD is essential before the onset of motor symptoms. Later on, motor symptoms appear while 50% of the dopaminergic neuron terminals in the *substantia nigra pars compacta*, and ~80% of the dopamine in the striatal are lost [5].

A variety of biological processes including RNA-based mechanisms regulate the expression of the genes. One of the main mechanisms of gene regulation is alternative splicing (AS). AS is a process that a single gene generates different types of transcripts and consequently expresses a number of protein isoforms with various functional characteristics [6]. AS is remarkably apparent in the central nervous system (CNS). Approximately expression of 40% genes in brain are regulated through AS [7]. Therefore, aberrant AS processing is a common feature of neurodegenerative disorders such as amyotrophic lateral sclerosis (ALS), Alzheimer's disease (AD), and PD [8]. In addition to monogenic types of PD which the following genes are involved: *SNCA* (NCBI accession no. 6622), *PARK2* (NCBI accession no. 5071), *PINK1* (NCBI accession no. 65018), *PARK7* (NCBI accession no. 11315), and *LRRK2* (NCBI accession no. 120892), other genes including *SRRM2* (Ensembl accession ENST00000301740.13), are detected to be pathogenic in PD due to their altered splicing regulation [9]. The *SRRM2*, RNA splicing factor, recognizes exon splicing enhancer sequences and promotes splicing by creating several essential interactions with factors directly bound to the pre-mRNA, such as the SR proteins family and U2 snRNP [10]. Differential AS pattern of *SRRM2* in PD patients' brain confirms that brain has a higher amount of AS processing than other tissues [11]. Accordingly *SRRM2* modulation in brain could be accounted as a biomarker of PD [12], but the regulatory mechanism and its expression in PD are still unknown. We suggest that a regulatory network of the genes are involved in splicing, under governing of *SRRM2*. Alternatively, post-transcriptional processing is regulated through miRNAs. Therefore, both miRNAs and AS may involve in PD onset [13, 14].

Blood cells, due to their specific cellular organization, reflect well to the physiological and pathological stimuli and treatments, as they link to the most tissues in the body such as the brain [15]. Interestingly, the transcriptomic patterns of the brain and peripheral blood mononuclear cells (PBMCs) overlap, about 80% [16]. Of note, blood-based biomarkers, which are minimally invasive, potentially used for diagnosis of PD. Such biomarkers are comprised proteins that exhibit differential expression [17], or other molecular elements, including miRNAs that are expressed by cells and tissues [18]. Therefore, assessment of miRNAs in PBMCs of PD patients could provide a valuable diagnosis tool to predict the progression and severity of PD [19].

In the present study, the expression of a gene along with multiple miRNAs were profiled in PBMCs of PD patients to candidate biomarkers for early diagnosis of PD. Utilizing *in-silico* analysis with a functional enrichment analysis for genes and miRNAs, we also attempted to understand more about the molecular etiopathology mechanisms of PD onset.

## Results

### *In-silico* studies and bioinformatics data

**Fig 1:**
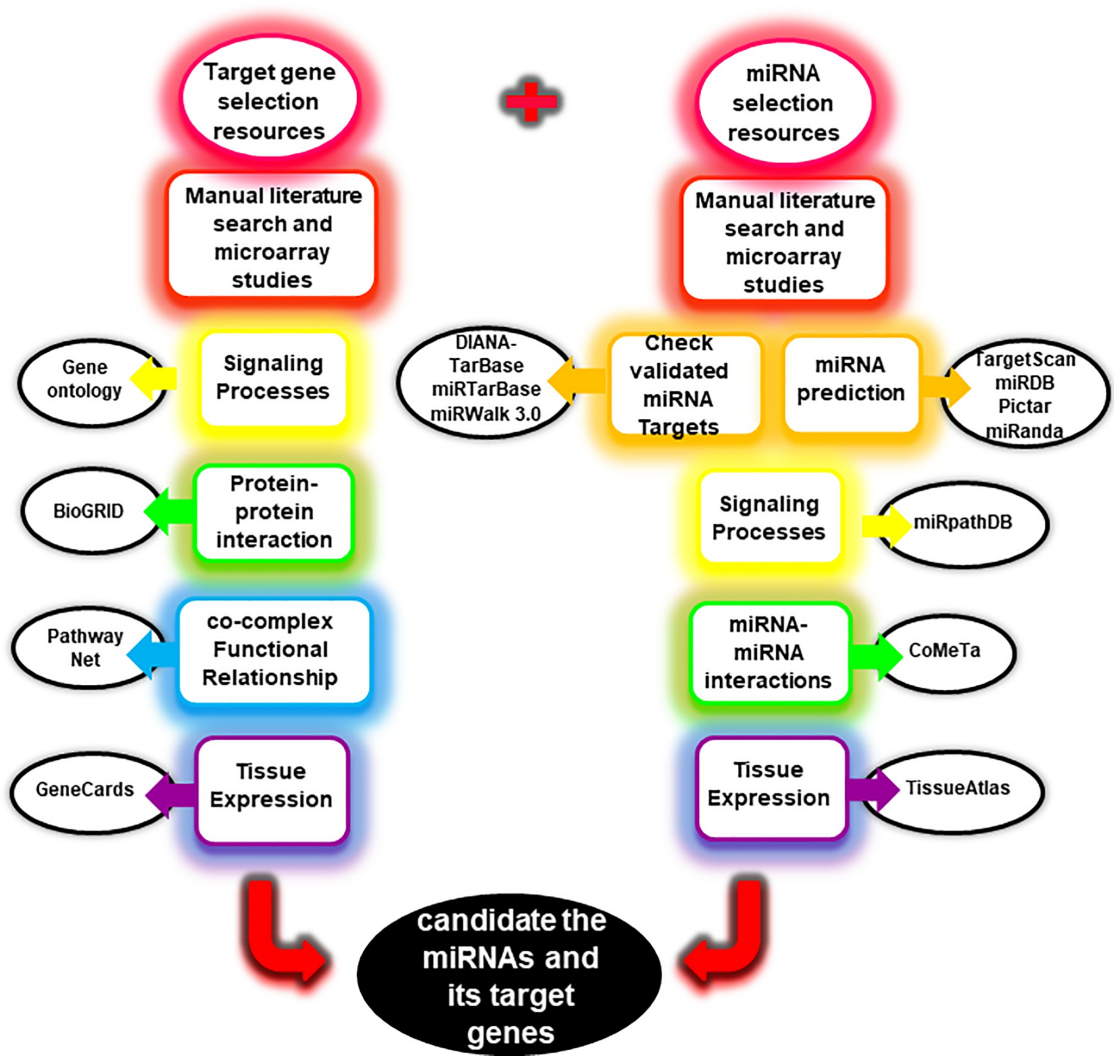
The resources for the selection of miRNAs and target gene. The general principles for selective resources of the miRNA-target gene are presented in the flowchart. Several databases and websites were used *in-silico* analysis of miRNAs and target gene. The names of databases and websites are displayed in non-colored elliptical forms. Details are mentioned in the text. The results of the *in-silico* analysis are presented in S1 Fig.

Candidate miRNAs and their target genes for this study were selected through the working flowchart as indicated (Fig 1). According to an analysis through gene ontology resources, *SRRM2* was identified to participates in several signaling pathways including spliceosomal complex (GO: 0005681, *p* = 1.09e-5) and catalytic step 2 spliceosome (GO: 0071013, *p* = 2.29e-7). Protein-protein interactions using BioGRID (S1A Fig) were used to identify other functional associated genes with *SRRM2*. Moreover a search for miR-27a/b-3p through miRPathDB, revealed a possible role of these miRs in mRNA splice site selection (GO: 0006376, *p* = 0.04). As shown in S1B Fig, through obtaining data of CoMeTa website, miR-27a-3p and miR-27b-3p have a functional relationship. The data of co-complex and functional relationships of PathwayNet indicated a network of associated genes with *SRRM2* (S1C and S1D Fig).

### miR-27a/b-3p target SRRM2 through binding to a conserved region among mammalian species

**Fig 2:**
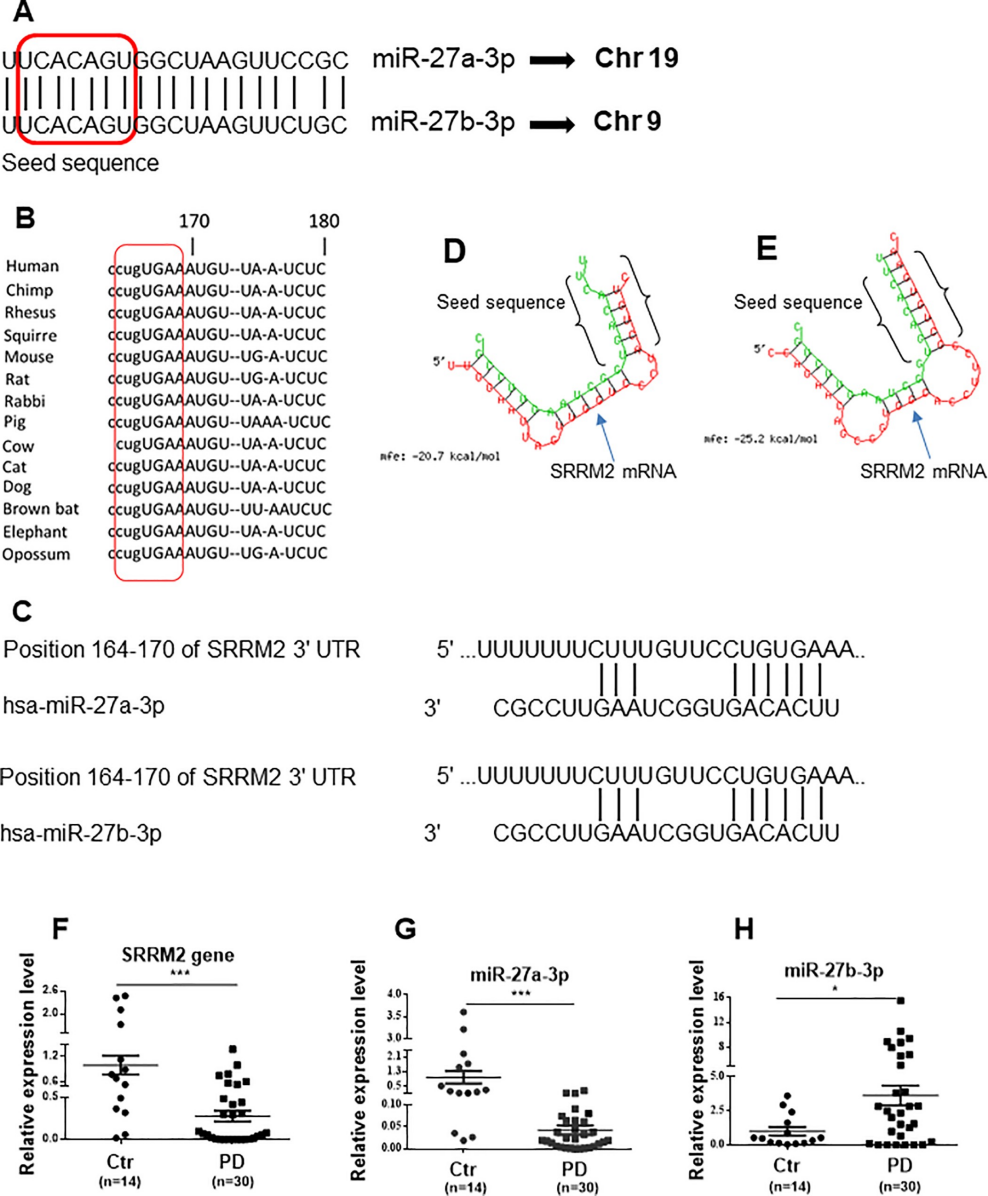
Expression levels and sequences of miR-27a/b-3p and *SRRM2*. **(A)** miR-27a-3p and miR-27b-3p share similar functional seed sequence with only one nucleotide difference. **(B)** A conserved region in *SRRM2* of several species responsible for binding to miR-27a-3p and miR-27b-3p. **(C)** The binding prediction of miR-27a-3p and miR-27b-3p to *SRRM2* into TargetScan is shown. **(D** and **E)** The minimum free energy (mfe) hybridization using the RNAhybrid database for miR-27a-3p and SRRM2 binding was shown. Downregulated expression of **(F)** *SRRM2* and **(G)** miR-27a-3p, and upregulated expression of **(H)** miR-27b-3p were determined in 30 PD patients and 14 healthy subjects using qRT-PCR analysis. Data are presented as means ± SEM. Differences were analyzed by two-tailed unpaired t-test in D, E and F. *p<0.05, *p<0.001 and ***p<0.001. Abbreviations: PD = Ctr = Control; PD = Parkinson's disease.

miR-27a/b-3p share similar mature sequences except for the only one nucleotide out of the seeding region (Fig 2A). The interacting region of *SRRM2*, with miR-27a/b-3p is conserved among mammalian species (Fig 2B) showing the importance of miR-27a/b-3p in post transcriptional regulation of *SRRM2*. The binding region of miR-27a/b-3p to human *SRRM2* transcript, using TargetScan, is predicted and shown in Fig 2C. The minimum free energy (mfe) hybridization using the RNAhybrid database for miR-27a-3p and SRRM2 binding was shown to be -20.7 kcal/mol through matching of five nucleotides out of 7 nucleotides of seed sequence, and mfe score for binding of miR-27b-3p to SRRM2 was predicted to be -25.2 kcal/mol as matched all seven nucleotides of seed sequence (Fig 2D and 2E).

### PBMCs levels of SRRM2 and miR-27a/b-3p are significantly modulated in PD patients

In this study, transcript levels of *SRRM2*, and miR-27a/b-3p were determined in PBMCs of 30 PD patients and 14 healthy subjects using qRT-PCR analysis. The demographic and clinical data of healthy subjects and PD patients who were recruited in this study are presented in Table 1.

**Table 1:** The demographic and clinical data collected from 30 Parkinson’s patients and 14 healthy controls.

	Controls	PD	Hoehn & Yahr stage 1	Hoehn & Yahr stage 2	Hoehn & Yahr stage 3	*p* Value^a^
**Number of Subjects**	14	30	11	11	8	-
**Age (Year)**	63.93±11.96	62±11.11	62±5.75	62±13.01	63±14.87	0.97
**Gender (F/M)**	3/11	9/21	4/7	2/9	3/5	0.43
**Disease duration (Month)**	-	68±65.89	39.59±39.68	55.4±52.9	132.29±78.44^c^	0.006
**UPDRS [Motor]**^**b**^	-	30±19.19	20±11.55	33±12.82	70±26.87^d^	0.0004

The data are presented as mean ± SD.

^a^Analysis of variance except for chi-square for gender.

^b^Off-state motor ratings according to the UPDRS.

^c^*p*<0.01 Hoehn & Yahr stage III group vs. Hoehn & Yahr stage I group.

^d^*p*<0.001 Hoehn & Yahr stage III group vs. Hohn & Yahr stage I group. Abbreviations: F/M = female/male; PD = Parkinson′s disease; UPDRS = Unified Parkinson′s disease rating scale.

PD patients were classified into stages 1–3 based on the severity of the disease (Hoehn-Yahr stage). Average age ± SD was for patients was 63.93 ± 11.96 years whilst it was 62 ± 11.11 years for control counterparts. The results of the expression of *SRRM2*, miR-27a/b-3p are presented in Fig 2F–2H that indicate a significant downregulation of *SRRM2* (*p* = 0.0002) and miR-27a-3p (*p* = 0.0001), and overexpression of miR-27b-3p (*p* = 0.02) in patients with PD compared to healthy controls, which were 0.28 ± 0.07, 0.04 ± 0.01 and 3.62 ± 0.62, respectively.

### Downregulation of miR-27a-3p was observed in all HY stages (disease severity), whereas the upregulation of miR-27b-3p was observed mostly at HY-1 and disease duration between 3–5 years

**Fig 3:**
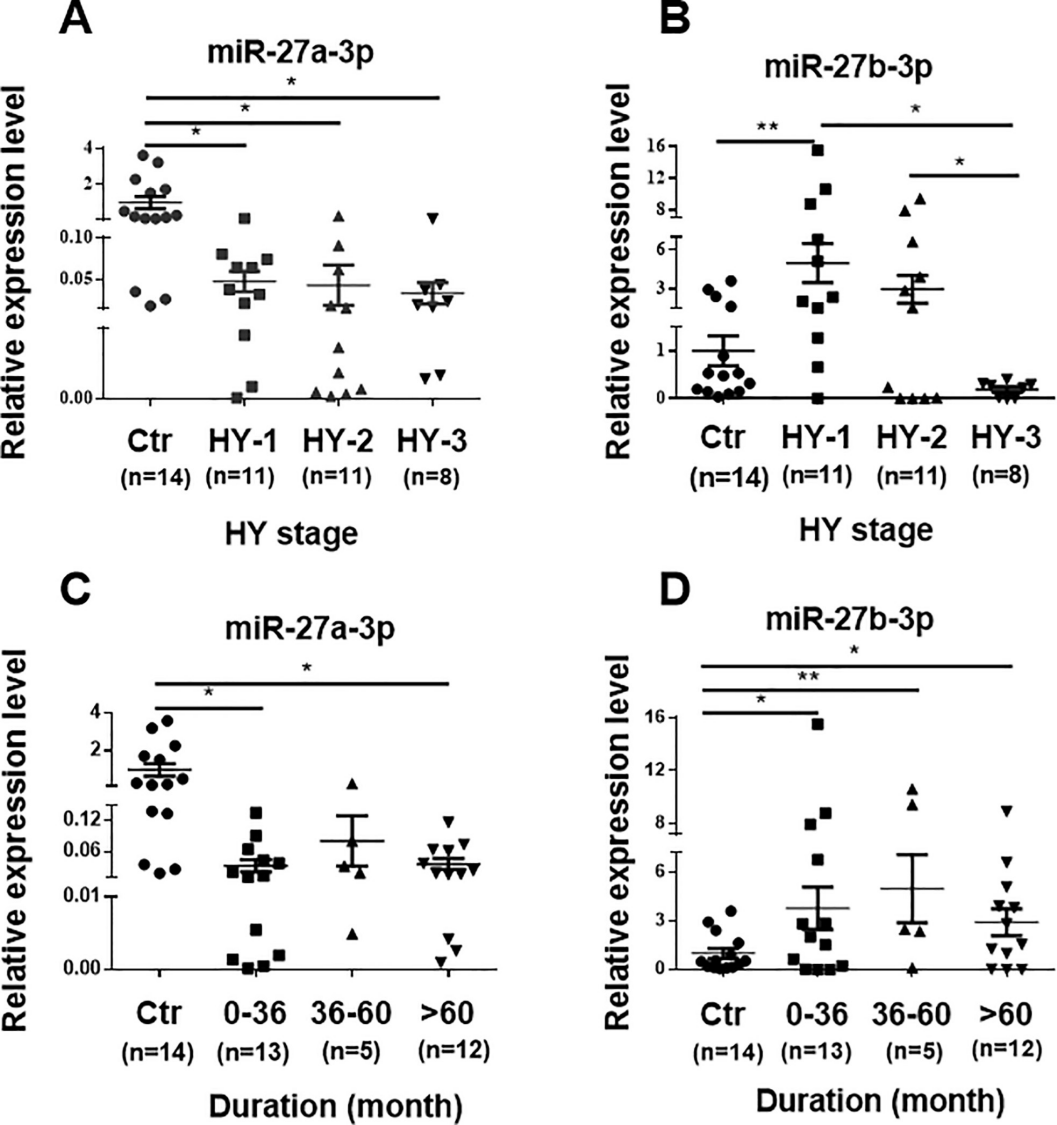
Correlation analysis of miR-27a/b-3p levels with disease severity and duration. **(A)** PBMCs derived miR-27a-3p level decreased along with the disease severity. **(B)** The expression of miR-27b-3p decreased with the disease severity, as was evidenced between HY-3 and HY-1 (*p* = 0.015) or HY-2 (*p* = 0.04) significantly. **(C)** Significant downregulation of miR-27a-3p expression with disease durations of 0–36 and >60 months **(D)** Upregulation of miR-27b-3p independent to disease duration. Data are presented as means ± SEM. Differences were analyzed by one-way ANOVA in A, B, C and D. *p<0.05, **p<0.001 and ***p<0.001. Ctr = Control; PD = Parkinson's disease patients; HY = Hoehn-Yahr.

A gradual decrease in miR-27a-3p levels was observed during progress of HY staging. The lowest expression of miR-27a-3p was detected between HY-3 patients and control subjects (Fig 3A). Despite descending wave of miR-27a-3p levels during progress of HY staging, there was no significant difference among HY stages presumably due to the small sample size of the patients (Fig 3A). There was a significant downregulation in miR-27a-3p during three stages of the illness, associated with increasing of disease severity.

There was a significant difference in the expression of miR-27b-3p in HY-1 patients as compared to controls (Fig 3B). Moreover, miR-27b-3p levels were decreased along with the disease severity, with a significant reduction in HY-3 vs. HY-1 and HY-3 vs. HY-2 (Fig 3B). Again, miR-27b-3p may predict disease severity and progression in PD. However, it appears that other molecular mechanisms may contribute to the decreasing trend of miR-27b-3p level along with HY staging.

The reduced amount of miR-27a-3p expression was apparent in PD patients′ PBMCs who underwent the illness diagnosis during 0–36 and >60 months significantly, whereas significant upregulation of miR-27b-3p expression was observed in all duration compare to control (Fig 3C and 3D). Accordingly, trend of miR-27a/b-3p expressions was not dependent to disease duration.

### miR-27a/b-3p levels showed an age-dependent wave of upregulation in healthy subjects dissimilar to PD patients

**Fig 4:**
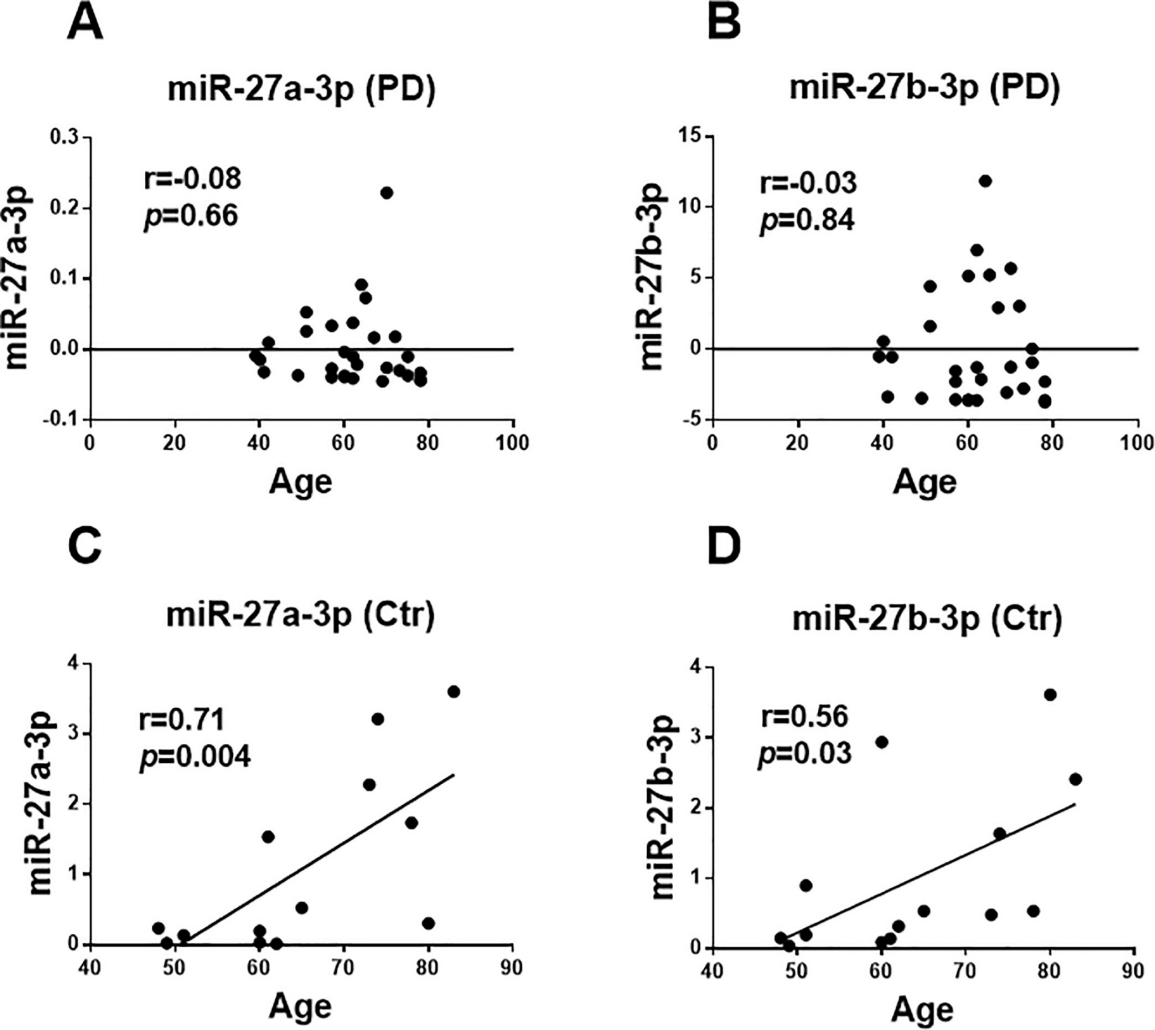
Association of miR-27a/b-3p levels with age factor. There are no relationships between expression of **(A)** miR-27a-3p and **(B)** miR-27b-3p with age of PD patients. Conversely, a direct significant relationship was observed in healthy subjects **(C)** for miR-27a-3p and **(D)** for miR-27b-3p association with age. Data were analyzed by Pearson’s correlation coefficient r and linear regression, with *p*-values are shown in the graphs. Ctr = Control; PD = Parkinson's disease patients.

There were no relationship between the expression of miR-27a-3p (r = -0.08, *p* = 0.66) and miR-27b-3p (r = -0.03, *p* = 0.84) with age in patients (Fig 4A and 4B). Contrariwise, a direct and significant relationship in healthy subjects for both miR-27a-3p (r = 0.71 and *p* = 0.004) and miR-27b-3p (r = 0.56 and *p* = 0.03) was obtained with the age (Fig 4C and 4D).

### A negative correlation was identified between the expression of SRRM2 and miR-27b-3p, whereas there was a positive correlation between miR-27a-3p and miR-27b-3p levels

**Fig 5:**
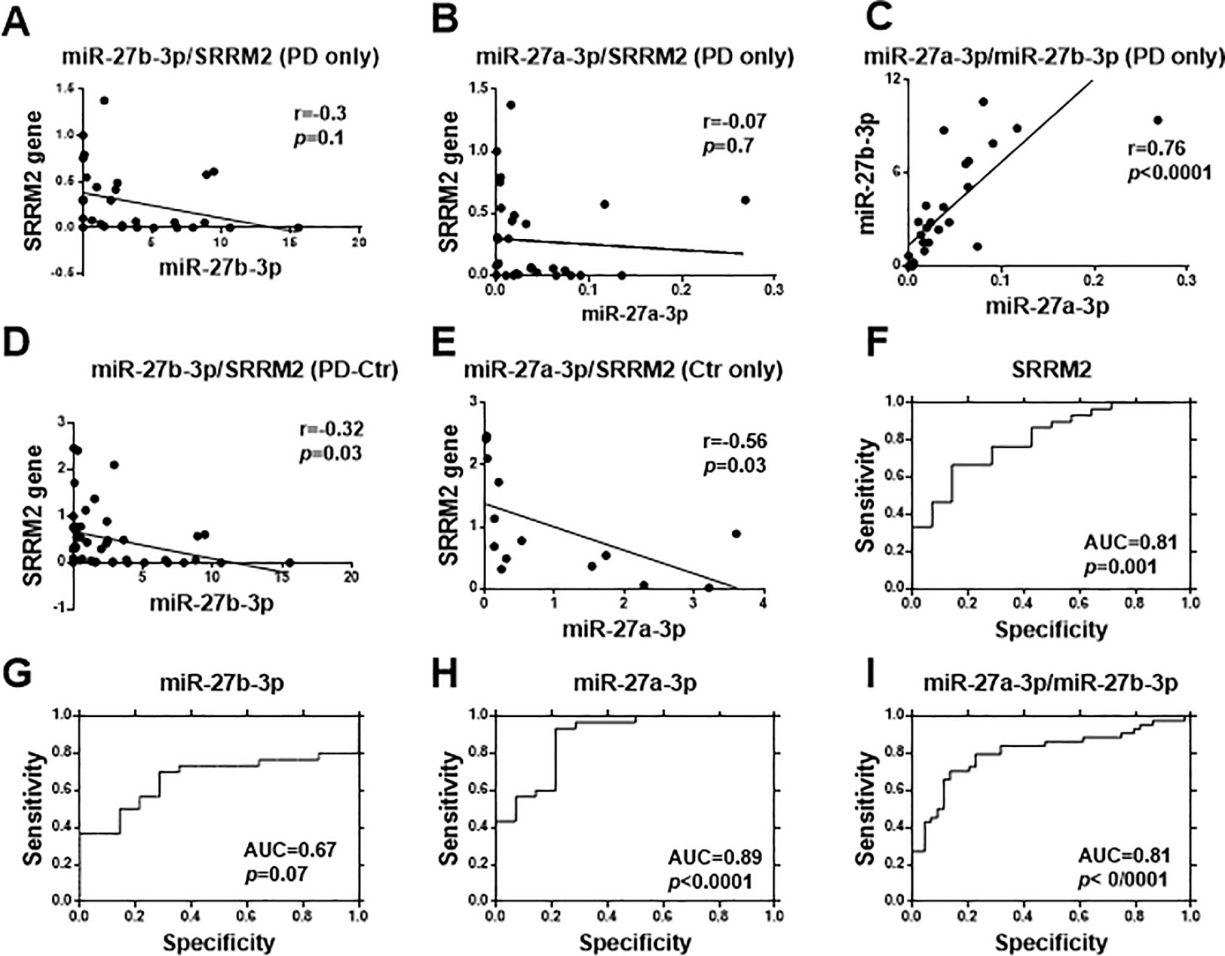
Correlation between *SRRM2* and miR-27a/b-3p levels, and their ROC curve. *SRRM2* expression correlate with **(A)** miR-27b-3p and **(B)** miR-27a-3p in PD patients, respectively. **(C)** miR-27a-3p and miR-27b-3p expressions correlate with together in PD patients. These accuracy for detection ability of PD were determined using the ROC curve. **(D)** Correlation between SRRM2/miR-27b-3p in PD patients vs. controls and **(E)** correlation between SRRM2/miR-27a-3p in healthy controls were presented. Their AUC were **(F)** 0.81 (95% CI: 0.57–0.98, p = 0.001), **(G)** 0.89 (95% CI: 0.5–0.95, P<0.0001), **(H)** 0.67 (95% CI: 0.57–0.98, p = 0.07) and **(I)** 0.81 (95% CI: 64.7–90.2, *p*<0.0001). Data about correlations were analyzed by Pearson’s correlation coefficient r and linear regression, with *p*-values are shown in the graphs. A summary of the correlation results in S1 Table and results of the ROC curve are presented in S2 Table. Abbreviations: Ctr = Control; PD = Parkinson disease.

Using Pearson's correlation coefficient and linear regression, significant inverse correlation (r = -0.3, *p* = 0.1, and r = -0.32, *p* = 0.03) between the expression of *SRRM2* and miR-27b-3p was revealed not only among the PD patients but also between PD patients and healthy subjects respectively (Fig 5A and 5D). Similar association (r = -0.07, *p* = 0.7, and r = -0.56, *p* = 0.03) was observed for expression levels of *SRRM2* and miR-27a-3p in PD patients and healthy subjects, respectively (Fig 5B and 5E). There was a direct correlation between miR-27a-3p and miR-27b-3p expression in PD patients (r = 0.76, p<0.0001) (Fig 5C). A summary of correlation results are presented in S1 Table.

### SRRM2 and miR-27a-3p are recommended as potential biomarkers for detection of PD

The levels of *SRRM2*, miR-27a-3p, miR-27b-3p, and miR-27a-3p/miR-27b-3p were evaluated for their biomarker ability of PD diagnosis. The biomarker accuracy of these parameters was determined through plotting the receiver-operating characteristic (ROC) curve. Their AUC (area under the ROC curve) were 0.81 (95% CI: 0.57–0.98, *p* = 0.001), 0.89 (95% CI: 0.5–0.95, *p*<0.0001), 0.67 (95% CI: 0.57–0.98, *p* = 0.07), and 0.81 (95% CI: 64.7–90.2, *p*<0.0001), respectively. Moreover, in cut-off values were 4.67 for *SRRM2*, 4.36 for miR-27a-3p, and 3.5 for miR-27b-3p, and 3.56 for miR-27a3p/miR-27b-3p. Also their sensitivity and specificity were 67%-86%, 93%-79%, 50%-86%, and 72.7%-79.6%, respectively (Fig 5F–5I). The results of the ROC curve are presented in S2 Table.

### The splicing genes appeared related to the pathways of toxic protein accumulation, the chaperone system, and the mitochondrial dysfunction

There was an association between the genes involved in splicing with the mitochondrial function genes, chaperone system and the inhibitor of the *α*-synuclein toxic protein accumulation (*TCP-1α*: NCBI accession 6950). To illustrate this relationship, a genetic network of 13 genes using the PathwayNet website (S2 Fig) was drawn. The genes that participate in the mitochondrial function and splicing processes had higher relationship confidence, such as *TCP-1α*, *SRSF2* (NCBI accession 6427), *PQBP1* (NCBI accession 10084), *PARK7*, *HNRNPA1* (NCBI accession 3178), *DNM1L* (NCBI accession 10059), and *SFPQ* (NCBI accession 6421). Other genes, including the *SRRM2*, *SNCA*, *PINK1*, and *PARK2*, had less relationship confidence.

### Functional enrichment analysis of the associated genes with SRRM2, miR-27a-3p, and miR-27b-3p indicated the shared biological processes and molecular functions

Functional enrichment analysis was carried out for a number of associated genes with *SRRM2* (Table 2).

**Table 2:** Functional enrichment analysis of *SRRM2* with 12 associated genes.

Category	KEGG pathway/ Gene ontology term	Genes	Number of genes	Fold enrichment*	*p*-value**	*p*-value^C^***
**GO_BP**	Protein stabilization	*PINK1*, *PARK7*, *PARK2*, *TCP-1α*	4	0.2	6.4E-5	0.004
**GO_CC**	Cytosol	*PINK1*, *PARK7*, *DNM1L*, *LRRK2*, *PARK2*, *SNCA*	6	0.3	7.1E-4	0.007
**GO_CC**	Nuclear speak	*PQBP1*, *SRSF2*, *SRRM2*, *PARK2*, *SFPQ*	5	0.3	6.2E-5	1.1E-5
**GO_CC**	Perinuclear region of cytoplasm/ Mitochondrion	*PINK1*, *DNM1L*, *PARK2*, *SNCA*	4	0.2	2.4E-3	0.028
**GO_MF**	Poly[A] RNA binding	*SRSF2*, *SRRM2*, *PARK7*, *SFPQ*, *TCP-1α*	5	0.2	0.007	0.042
**KEGG**	Parkinson's disease	*PINK1*, *PARK7*, *LRRK2*, *PARK2*, *SNCA*	5	0.2	2.4E-5	1.1E-5
**KEGG**	Spliceosome	*HNRNPA1*, *PQBP1*, *SRSF2*, *SRRM2*	4	0.2	5.7E-4	1.25E-4

Significantly enriched GO annotations and KEGG pathways were obtained using the DAVID bioinformatics resources. Fold Enrichment* is presented in terms of percentage. *p*-value** was calculated using the Fisher Exact test. *p*-value^c^*** after Benjamini-Hochberg correction. Abbreviations: KEGG = KEGG Pathway; GO_BP = Gene Ontology, Biological Process; GO_CC = Gene ontology, Cellular component; GO_MF = Gene ontology, Molecular function.

The products of *PQBP1*, *SRSF2*, *SRRM2*, and *HNRNPA1* are involved in the spliceosome pathway. On the other hand, products of *PINK1*, *PARK2*, *TCP-1α*, and *PARK7* participate in the biological process of protein stabilization. Indeed, all products of those genes associating with *SRRM2* were shown to be involved in traffic pathway between nucleus and mitochondria. The genes involved in Poly(A) RNA binding include *SRSF2*, *SRRM2*, *PARK7*, *TCP-1α*, and *SFPQ*, which their respective proteins participate in splicing, mitochondrial function, and chaperone system and protein folding.

To delineate whether miR-27a/b-3p are involved in same pathways, comprehensive functional enrichment analysis carried out using GO enrichment and KEGG pathways, respectively (S3A and S3B Fig). As shown in Table 3, a number of those significant pathways are mentioned.

**Table 3:** Functional enrichment analysis of miR-27a-3p and miR-27b-3p.

Category	KEGG Pathway/Gene Ontology term	*p*-value*	miRNAs
**GO_BP**	mitotic cell cycle (GO:0000278)	<1e-325	2
**GO_BP**	transcription, DNA-templated (GO:0006351)	<1e-325	2
**GO_BP**	protein complex assembly (GO:0006461)	<1e-325	2
**GO_BP**	cellular protein modification process (GO:0006464)	<1e-325	2
**GO_BP**	response to stress (GO:0006950)	<1e-325	2
**GO_BP**	epidermal growth factor receptor signaling pathway (GO:0007173)	<1e-325	2
**GO_BP**	blood coagulation (GO:0007596)	<1e-325	2
**GO_BP**	cell death (GO:0008219)	<1e-325	2
**GO_BP**	catabolic process (GO:0009056)	<1e-325	2
**GO_BP**	biosynthetic process (GO:0009058)	<1e-325	2
**GO_BP**	gene expression (GO:0010467)	<1e-325	2
**GO_BP**	viral process (GO:0016032)	<1e-325	2
**GO_BP**	cellular component assembly (GO:0022607)	<1e-325	2
**GO_BP**	cellular nitrogen compound metabolic process (GO:0034641)	<1e-325	2
**GO_BP**	nucleobase-containing compound catabolic process (GO:0034655)	<1e-325	2
**GO_BP**	Fc-epsilon receptor signaling pathway (GO:0038095)	<1e-325	2
**GO_BP**	post-translational protein modification (GO:0043687)	<1e-325	2
**GO_BP**	cellular protein metabolic process (GO:0044267)	<1e-325	2
**GO_BP**	small molecule metabolic process (GO:0044281)	<1e-325	2
**GO_BP**	symbiosis, encompassing mutualism through parasitism (GO:0044403)	<1e-325	2
**GO_BP**	neurotrophin TRK receptor signaling pathway (GO:0048011)	<1e-325	2
**GO_BP**	membrane organization (GO:0061024)	<1e-325	2
**GO_BP**	macromolecular complex assembly (GO:0065003)	<1e-325	2
**GO_BP**	protein complex (GO:0043234)	<1e-325	2
**GO_BP**	mRNA processing (GO:0006397)	2.386361E-05	2
**GO_BP**	RNA splicing (GO:0008380)	9.596125E-05	2
**GO_BP**	negative regulation of translation involved in gene silencing by miRNA (GO:0035278)	0.0271003	2
**GO_BP**	regulation of mRNA stability (GO:0043488)	0.02790592	2
**GO_CC**	nucleoplasm (GO:0005654)	<1e-325	2
**GO_CC**	cytosol (GO:0005829)	<1e-325	2
**GO_CC**	organelle (GO:0043226)	<1e-325	2
**GO_B**	protein complex (GO:0043234)	<1e-325	2
**GO_MF**	protein binding transcription factor activity (GO:0000988)	<1e-325	2
**GO_MF**	nucleic acid binding transcription factor activity (GO:0001071)	<1e-325	2
**GO_MF**	RNA binding (GO:0003723)	<1e-325	2
**GO_MF**	cytoskeletal protein binding (GO:0008092)	<1e-325	2
**GO_MF**	enzyme binding (GO:0019899)	<1e-325	2
**GO_MF**	ion binding (GO:0043167)	<1e-325	2
**GO_MF**	poly(A) RNA binding (GO:0044822)	<1e-325	2
**KEGG**	Fatty acid synthesis (hsa00061)	<1e-325	2
**KEGG**	Prion diseases (hsa05020)	<1e-325	2

Significantly GO annotations, and KEGG pathways were obtained using the DIANA-mirPath. The most significant results are presented. *p*-value* was calculated using the Fisher Exact test. The comprehensive functional enrichment analysis of miR-27a-3p and miR-27b-3p are presented in S3A and S3B Fig. Abbreviations: KEGG = KEGG Pathway; GO_BP = Gene ontology, Biological Process; GO_CC = Gene ontology, Cellular component; GO_MF = Gene ontology, Molecular function.

miR-27a/b-3p are involved in various biological processes, including mRNA processing, RNA splicing, and protein processing, and molecular functions such as RNA binding and poly(A) RNA binding (S3A Fig). Among KEGG signaling pathways, fatty acid synthesis and prion disease pathways were most significant pathways (S3B Fig).

## Discussion

Early diagnosis of PD is an important issue to prohibit the progress of disease and reduces both symptoms and cost of treatment [20]. The usage of blood cells as a valuable resource for genetic biomarkers in neurological conditions was first reported in 1975 [21]. PBMCs could be obtained at each stage of the PD, whereas dopaminergic neurons could be accessible from the post-mortem brain of PD patients. Most disturbances of biological pathways in PBMCs embody the pathologic state of PD patients′ brains [22]. Therefore, it is possible to use PBMCs as valuable samples for further studies [23]. Perturbations in immune functions of PD patients have been reported already [24]. These perturbations include variations in T lymphocyte subset, a weak response of PBMCs to mitogens, and the impaired production of IL-2 by the PBMCs [24]. Whether these immunologic primary disturbances, induce neuronal cell death, are still unclear. In patients with PD, CNS dysfunction may cause changes in the immune function of PBMCs, vice versa the direct activation of the immune system may advocate neuronal dysfunction [25]. Therefore, the study of PBMCs changes in PD may better interpret etiology of the impaired pathways and disease.

Expression of *SRRM2* has already been assessed by Shehadeh et al. [12] as showed a downregulation in long transcript of *SRRM2* in *substantia nigra* in a good agreement with both our data on PBMCs in this study and a microarray study on PD patients′ PBMCs [GEO Accession GSE18838] PD patients [12]. Such similarity reflects presence of the common regulatory mechanisms of *SRRM2* between PBMCs and brain tissue.

miR-27a/b-3p were downregulated as reported by a microarray study on miRNAs derived from PBMCs of PD patients [GEO Accession GSE16658] [54]. On the contrary, Cogswell et al. showed that miR-27a/b-3p are upregulated in the hippocampus and medial frontal gyrus brain areas of AD patients subjects [26]. Butovsky et al. indicated that miR-27a/b-3p expression in peripheral monocytes of ALS patients were upregulated [27], whereas Sørensen showed that the level of these miRs decreased in cerebrospinal fluid (CSF) of AD patients [28]. Such discrepancy of reports indicates distinct tissue-specific regulatory mechanisms for miR-27a/b-3p levels. Similar reduction in miR-27a-3p level is reported in Huntington's disease, traumatic brain injury (TBI). It is supposed that increased levels of miR-27a-3p may exert protective effects on brain as it act against neuronal death and blood-brain barrier (BBB) permeability and autophagy activation after TBI [29]. Interestingly, the expression level of miR-27b-3p is upregulated in CSF, blood, and saliva of patients with TBI [30]. Recently, Ma et al. have shown that both in the wild type and *ob/ob* mice, isomiRs in mmu-miR-27b-3p is more expressed than those in mmu-miR-27a-3p. They also reported that in the small RNA sequencing data obtained from the TCGA database in human liver cancer, miR-27b-3p expressed more than miR-27a-3p [31]. The expression of miR-27a/b-3p in PD patients did not correlate with the age factor, but there was a direct significant correlation between these parameters in healthy subjects. Accordingly, Chen et al. found that miR-23-27-24 family levels were raised in the mouse brain cortex during the increasing of age. Also at same developmental periods, increased expression of miR-23b-27b cluster in adult mice vs. E18 stage was apparent than miR-23a-27a cluster. In fact, an increase in the expression of this family during the development of the brain causes downregulation of the target gene, *apoptotic protease activating factor-1* (*Apaf-1*), to trigger protection of the brain neurons against apoptosis [32]. Inconsistently, Eisenberg et al. indicated that spermidine, a crucial factor for inhibiting aging through epigenetic modifications, autophagy induction, and the inhibition of necrosis [33] is a target of miR-27a-3p [34]. However, our results on miR-27a/b-3p is in good agreement with the former study.

Negative regulation of miRNA on gene expression often shows an inverse correlation between miRNA and its target. The negative correlation coefficient may suggest an effective way to identify miRNA-target pair [35]. Accordingly there was a non-significant and very low negative correlation between miR-27a-3p and *SRRM2* in PD patients, delineating that miR-27a-3p may not perform a role in regulating *SRRM2* in PD. There was also a negative correlation between miR-27b-3p and *SRRM2* among in PD patients, but not significant, however it was significant between PD patients and healthy subjects elucidating that miR-27b-3p may negatively regulate *SRRM2* in PD.

Positive miRNA-miRNA correlation is a reflect of feed-forward regulation through the act of transcription factors, whereas negative miRNA-mRNA correlation is triggered by miRNA inhibitory effect on target gene [36]. Coordinated interactions between miRNAs of a cluster, which often show a high correlation in their expression profiles, may regulate biological processes [37, 38], although they may exhibit different levels of enrichment due to maturation and degradation mechanisms. When a target mRNA is shared for the two miRNAs, a complicate regulatory pattern may emerge [37]. For instance, one miRNA can affect the expression level of its partner miRNA through regulating their common target gene, thereby monitors the activity of its partner miRNA [39]. Such viewpoint could be extended to miR-27b-3p which regulates both levels of miR-27a-3p and *SRRM2* which are downregulated in PD patients.

Both the *SRRM2*, a member of spliceosome complex and miR-27a/b-3p contribute to the splicing process. Genes associated with SRRM2 (part 8 of results) are contributing the pathways of protein stabilization and splicing. Distribution of target genes in the traffic between nuclear speak, cytosol and mitochondrion indicate a correlation between the pathways of protein accumulation and folding, splicing, and mitochondrial function.

Using the miRpathDB database, we also found that both miR-27a-3p and miR-27b-3p are significantly involved in the mRNA splice site selection (GO: 0006376) in a good agreement with previous study showing miR-27a-3p relation to the spliceosome pathway [40]. On the other hand, using KEGG database, the relationship of miR-27a with AD, PD, and brain malignancies has been identified [41]. Eventually, similarities between the functional enrichment analysis of the genes mentioned above and miR-27a/b-3p suggest that they act in common functional pathways, and their dysfunction communicates to molecular etiopathology mechanisms of PD onset.

## Conclusion

Taken together, combination of miR-27a-3p and *SRRM2* levels could be considered as valuable biomarker for PD as their expression levels are downregulated in PD patients.

## Materials and methods

### Ethical issue

All protocols for the usage of human samples in this study were reviewed and approved by Institutional Review Board of Royan Institute (IR.ACECR.ROYAN.REC.1397.80) in accordance with the relevant guidelines and regulations for using of human samples. Moreover, written informed consents were obtained from all participants according to the guidance of Institutional Review Board.

### Patients

Thirty PD patients were recruited from Parkinson's Clinic of Al-Zahra Hospital (Isfahan, Iran). PD cases were clinically diagnosed by a neurologist, according to the UK brain bank criteria [42]. Exclusion criteria were hereditary PD, clinical symptoms of atypical Parkinsonism, psychiatric disorders, systemic diseases, such as diabetes. The patients′ stage and severity of motor symptoms were assessed using modified Hoehn and Yahr (HY) stage [43], and movement disorder society-unified Parkinson’s disease rating score (MDS-UPDRS) [44], respectively. Fourteen age and gender matched volunteer healthy subjects without any neurological and psychiatric symptoms were recruited after verifying by a neurologist.

### Blood sampling and PBMCs isolation

We took 5 mL of the peripheral blood of each patient and healthy subject. Samples were collected in 15 mL centrifuge tubes (SPL Life Sciences, Pocheon-si, Korea) and PBMCs were extracted using Lymphodex (Inno-train Diagnostik GmbH, Kronberg im Taunus, Germany) and Ficoll-Paque density gradient centrifugation at 200×g for 20 minutes. The protocol of isolation of mononuclear cells from whole blood using density gradient centrifugation is available at http://dx.doi.org/10.17504/protocols.io.4v3gw8n [PROTOCOL DOI].

### RNA extraction, quantity and quality assessment

Total RNA from PBMCs was extracted using TRIzol Reagent (Thermo Fisher Scientific, Waltham, MA, USA). The protocol of RNA extraction using TRIzol reagent is available at http://dx.doi.org/10.17504/protocols.io.iykcfuw [PROTOCOL DOI]. The quantity and quality assessment of extracted RNAs were performed using agarose gel electrophoresis and NANODROP 2000c spectrophotometer (Thermo Fisher Scientific), respectively.

### Reverse transcription and quantitative real-time PCR (qRT-PCR)

Initially, 1 μg of total RNA was used for the synthesis of the first strand of cDNA using PrimeScript 1st strand cDNA Kit (TaKaRa Bio, Kusatsu, Shiga, Japan) and 500 ng of total RNA for the synthesis of the first strand micro cDNA using Universal cDNA Synthesis kit II, 8–64 rxns (Exiqon, Vedbæk, Denmark), respectively. qRT-PCR was achieved using QuantiFast SYBR Green PCR kit (QIAGEN, Hilden, Germany). The relative expression level of mRNAs and miRNAs were calculated using the 2^-ΔCt^ method [45]. *GAPDH* (NCBI accession no. 2597) and *β-ACTIN* (NCBI accession no. 60), and miR-191-5p (miRBase accession no. MIMAT0000440) and *U6 snRNP* (NCBI accession no. 26827) were used as endogenous controls for normalizing of mRNA and miRNA expression levels, respectively. *GAPDH* and *β-ACTIN* were already reported to be used for the neurological studies [46], *U6* is a universal reference for miRNA RT-qPCR studies [47], and miR-191-5p was also reported to be used for qRT-PCR of blood samples [48] and was introduced as a reliable endogenous control by the Exiqon company (miRCURY LNA Universal RT microRNA PCR system). Normalization was performed using geometric averaging [49] of miR-191-5p and *U6 snRNP*, *GAPDH*, and *β-ACTIN* expressions.

### Prediction of functional gene network

Gene symbols were entered to the PathwayNet website [50], and the functional gene network was visualized for *SRRM2* and 12 respective genes.

### Functional enrichment analysis of genes and miRNAs

Functional enrichment analyses of genes and miRNAs were obtained using the DAVID Bioinformatics Resources (RRID:SCR_001881) [51] and DIANA-mirPath [52], respectively. Functional enrichment analysis of miRNAs was addressed as heatmap images in S3A and S3B Fig.

### Statistical analysis

Data were presented based on the mean ± SEM. Comparisons between two and more groups were carried out using the two-tailed unpaired *t*-test and one-way ANOVA, respectively. The correlation between miR-27a/b-3p expression with age, miRNAs expression with their target genes, and miRNAs expression with each other were estimated using Pearson's correlation coefficient and linear regression. The receiver operating characteristic (ROC) curve was plotted using GraphPad PRISM 6.0 (RRID: SCR_002798, La Jolla California, USA). Statistical analysis was accomplished using GraphPad PRISM 6.0 and IBM SPSS statistics 23 (RRID: SCR_002865, Armonk, New York, USA). Furthermore, *p* < 0.05 was defined as statistically significant difference between variables.

### The resources for the selection of miRNAs and their target genes

Candidate miRNAs and their target genes for experimental studies were selected through the working flowchart as indicated (Fig 1). The *SRRM2* was selected from the Shehadeh et al. study [12] and microarray data with GEO Accession: GSE6613, relates to cellular blood of Parkinson's patients and is available from GEO DataSets portal [53]. The miRNAs involved in the PBMCs of Parkinson's patients were obtained from microarray data with GEO Accession: GSE16658 [54]. To predict miRNAs that target *SRRM2*, we used the TargetScan (RRID: SCR_010845, target score for miR-27a/b-3p: Context++ score: -0.08, and Context++ score percentile: 77) [55], miRDB (RRID: SCR_010848, target score for miR-27a/b-3: 71) [56], Pictar (RRID: SCR_003343, Pictar score for miR-27a/b-3p: 3.19, probabilities: 0.96, and free energies: -17.1 kcal/mol) [57], and miRanda (RRID: SCR_006997, mirSVR score for miR-27a/b-3p: -0.29) [58]. Using the DIANA-TarBase (RRID: SCR_010841) [59], miRTarBase [60], miRWalk 3.0 (RRID: SCR_016509) [61], it was determined that the SRRM2 for miR-27a/b-3p had not yet been validated with methods of less-strong and strong evidence. Signaling pathways of the *SRRM2* were acquired using Gene Ontology Resource [62] and miRNAs by miRPathDB [63]. Protein-protein interaction data for *SRRM2* were provided using BioGRID (RRID: SCR_007393) [64] and miRNA-miRNA interaction data for miR-27a-3p (miRBase accession MIMAT0000084) and miR-27b-3p (miRBase accession MIMAT0000419) provided using the CoMeTa website [65]. Information related to co-complex and functional relationships for *SRRM2* were obtained using the PathwayNet [50]. The minimal free energy (mfe) hybridization between miR-27a/b-3p and SRRM2 was obtained using the RNAhybrid database [66] (RRID:SCR_003252) (Fig 2D and 2E). Finally, *SRRM2* had the expression in nervous and immune cells, was obtained from the GeneCards (RRID: SCR_002773) [67] and miR-27a/b-3p in body fluids, including blood cells, were obtained from the TissueAtlas [68]. The results of the *in-silico* analysis are presented in S1 Fig.

## Supporting information

S1 Fig The results of *in-silico* analysis of miRNA and target gene. **(a)** Protein-protein interaction for *SRRM2* were provided from BioGRID and **(b)** miRNA-miRNA interaction for miRNAs using the CoMeTa website. Information related to **(c)** co-complex, **(d)** functional relationships, and for *SRRM2* were obtained using the PathwayNet. **(e)** *SRRM2* expression in body tissues from the GeneCards and **(f** and **g)** for the miR-27a-3p and miR-27b-3p in the body biofluids was obtained from the TissueAtlas, respectively.
(TIF)

S2 Fig Gene networks of *SRRM2*-associated partner genes. These gene networks was visualized using the PathwayNet to determine whether genes interact with each other. It is evident that a number of genes has more interactions with higher relationship confidence, including those involved in the mitochondrial function and splicing process, such as *TCP1*, *SRSF2*, *PQBP1*, *PARK7*, *HNRNPA1*, *DNM1L*, and *SFPQ*. The functional relationship with less confidence belonged to the *SRRM2*, *SNCA*, *PINK1*, and *PARK2*. *LRRK2* had the least functional relationship with partner genes. The red color line shows higher level of relationship confidence while blue color line indicates less relationship confidence.
(TIF)

S3 Fig The comprehensive functional analysis of miR-27a-3p and miR-27b-3p. **(a)** Significantly GO annotations and **(b)** KEGG pathways were obtained using the DIANA-mirPath. The most significant functions are shown with red color whereas cream color is used for those of less functional significance.
(TIF)

S1 Table Correlation coefficient between *SRRM2*, miR-27a-3p, and miR-27b-3p. Data were analyzed by Pearson’s correlation coefficient r and linear regression. p-values are shown in the Table. PD and Ctr represent Parkinson′s disease patient and control individuals respectively.
(PDF)

S2 Table The accuracy and results of ROC curve for *SRRM2*, miR-27a-3p, and miR-27b-3p for detection ability of PD from healthy controls. AUC = area under curve.
(PDF)

S1 File The values used to build graphs. (DOCX)

## Abbreviations

AD: Alzheimer’s disease
ALS: amyotrophic lateral sclerosis
AS: alternative splicing
AUC: area under the ROC curve
BBB: blood-brain barrier
CNS: central nervous system
CSF: cerebrospinal fluid
HY: Hoehn and Yahr
ICH: intracerebral hemorrhage
MDS-UPDRS: Movement disorder society-unified Parkinson’s disease rating score
MFE: Minimum free energy
PBMCs: peripheral blood mononuclear cells
PD: Parkinson’s disease
qRT-PCR: quantitative real-time PCR
ROC: receiver operating characteristic
TBI: traumatic brain injury
UPDRS: Unified Parkinson’s disease rating score
