# Correction: A compound downregulation of *SRRM2* and miR-27a-3p with upregulation of miR-27b-3p in PBMCs of Parkinson’s patients is associated with the early stage onset of disease

**DOI:** 10.1371/journal.pone.0244776

**Published:** 2020-12-30

**Authors:** Soudabeh Fazeli, Majid Motovali-Bashi, Maryam Peymani, Motahare-Sadat Hashemi, Masoud Etemadifar, Mohammad Hossein Nasr-Esfahani, Kamran Ghaedi

There is an error in affiliation 2 for author Maryam Peymani. The correct affiliation 2 is: Department of Biology, Faculty of Basic Sciences, Shahrekord Branch, Islamic Azad University, Shahrekord, Iran.
