# Supplementary figures and images for: A compound downregulation of SRRM2 and miR-27a-3p with upregulation of miR-27b-3p in PBMCs of Parkinson’s patients is associated with the early stage onset of disease
Source: PLoS One. 2020 Nov 10;15(11):e0240855. doi: 10.1371/journal.pone.0240855 (PMC7654768; doi:10.1371/journal.pone.0240855)

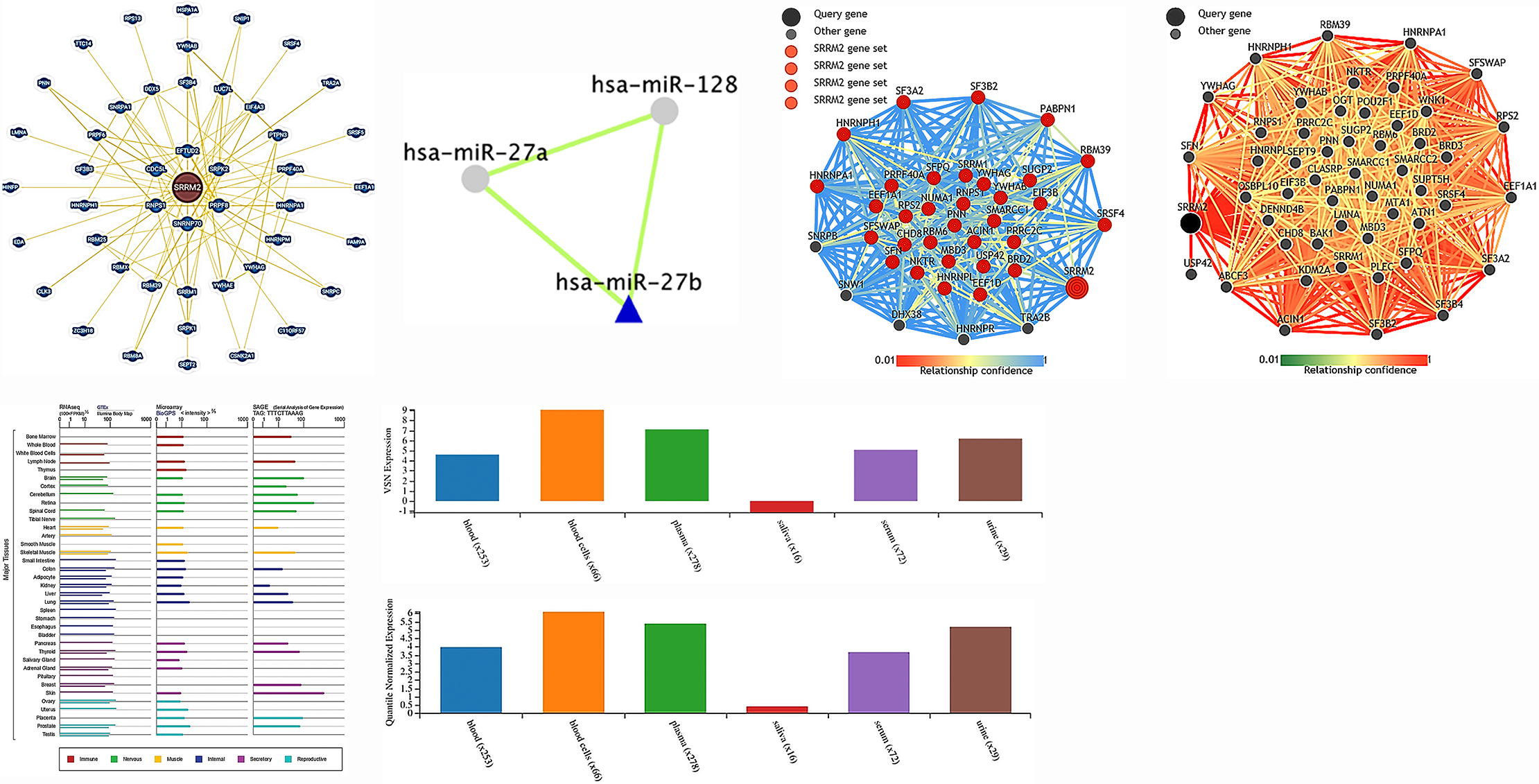

Supplement: S1 Fig — (a) Protein-protein interaction for SRRM2 were provided from BioGRID and (b) miRNA-miRNA interaction for miRNAs using the CoMeTa website. Information related to (c) co-complex, (d) functional relationships, and for SRRM2 were obtained using the PathwayNet. (e) SRRM2 expression in body tissues from the GeneCards and (f and g) for the miR-27a-3p and miR-27b-3p in the body biofluids was obtained from the TissueAtlas, respectively. (TIF) [file pone.0240855.s001.tif]

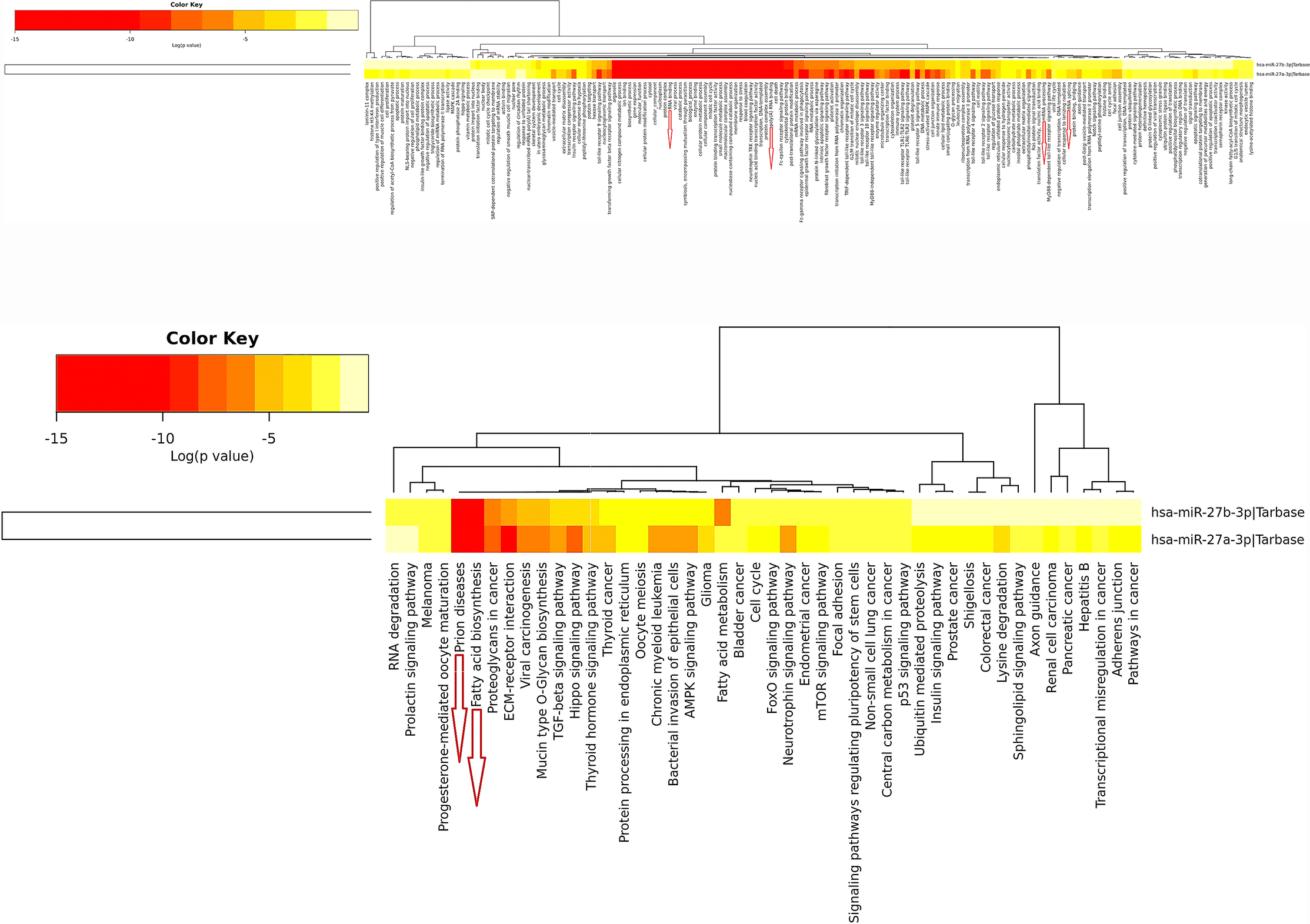

Supplement: S3 Fig — (a) Significantly GO annotations and (b) KEGG pathways were obtained using the DIANA-mirPath. The most significant functions are shown with red color whereas cream color is used for those of less functional significance. (TIF) [file pone.0240855.s003.tif]
