# Supplementary material for: A compound downregulation of SRRM2 and miR-27a-3p with upregulation of miR-27b-3p in PBMCs of Parkinson’s patients is associated with the early stage onset of disease
Source: PLoS One. 2020 Nov 10;15(11):e0240855. doi: 10.1371/journal.pone.0240855 (PMC7654768; doi:10.1371/journal.pone.0240855)
Supplement: S1 Table — Data were analyzed by Pearson’s correlation coefficient r and linear regression. p-values are shown in the Table. PD and Ctr represent Parkinson′s disease patient and control individuals respectively. (PDF) [file pone.0240855.s004.pdf]

S1 Table. Correlation coefficient between *SRRM2*, miR-27a-3p, and miR-27b-3p

miRNA/Gene	Pearson correlation	*p*-value
SRRM2 / miR-27b-3p (PD)	r=-0.3	0.1
SRRM2 / miR-27a-3p (PD)	r=-0.07	0.7
miR-27a-3p/ miR-27b-3p (PD)	r=0.76	1E-4
SRRM2 / miR-27b-3p (PD-Ctr)	r=-0.32	0.03
SRRM2 / miR-27a-3p (Ctr)	r=-0.56	0.03

Data were analyzed by Pearson's correlation coefficient r and linear regression. *p*-values are shown in the Table. PD and Ctr represent Parkinson′s disease patient and control individuals respectively.
