# Supplementary material for: A compound downregulation of SRRM2 and miR-27a-3p with upregulation of miR-27b-3p in PBMCs of Parkinson’s patients is associated with the early stage onset of disease
Source: PLoS One. 2020 Nov 10;15(11):e0240855. doi: 10.1371/journal.pone.0240855 (PMC7654768; doi:10.1371/journal.pone.0240855)
Supplement: S2 Table — AUC = area under curve. (PDF) [file pone.0240855.s005.pdf]

S2 Table. The accuracy and results of ROC curve for *SRRM2*, miR-27a-3p, and miR-27b-3p for detection ability of PD from healthy controls.

miRNA/Gene	Cut-off value	Sensitivity (%)	Specificity (%)	Accuracy (%) (AUC)	*p*-value
SRRM2	4.67	67	86	0.81	0. 001
miR-27a-3p	4.36	93	79	0.89	<0.0001
miR-27b-3p	3.5	50	86	0.67	0.07
miR-27a-3p/miR-27b-3p	3.56	72.7	79.6	0.81	<0.0001

AUC= area under curve.
