# Supplementary material for: A compound downregulation of SRRM2 and miR-27a-3p with upregulation of miR-27b-3p in PBMCs of Parkinson’s patients is associated with the early stage onset of disease
Source: PLoS One. 2020 Nov 10;15(11):e0240855. doi: 10.1371/journal.pone.0240855 (PMC7654768; doi:10.1371/journal.pone.0240855)
Supplement: S1 File — (DOCX) [file pone.0240855.s006.docx]

1. **The values used to build graphs that related to: expression levels of SRRM2 and miR-27a.b-3p (Fig 2).**

| **SRRM2** | | |
| --- | --- | --- |
| **NO.** | **PD** | **Ctr** |
| **1** | 0.008764 | 2.107179 |
| **2** | 0.575051 | 2.418022 |
| **3** | 0.307795 | 0.318271 |
| **4** | 0.003636 | 0.017666 |
| **5** | 0.077985 | 0.891364 |
| **6** | 0.292859 | 0.540961 |
| **7** | 0.545028 | 0.058729 |
| **8** | 1.002872 | 0.779865 |
| **9** | 0.754366 | 1.722561 |
| **10** | 0.098385 | 1.132863 |
| **11** | 0.440114 | 0.685435 |
| **12** | 0.486236 | 0.365083 |
| **13** | 1.378699 | 0.492398 |
| **14** | 0.793084 | 2.469601 |
| **15** | 0.609729 |  |
| **16** | 0.001933 |  |
| **17** | 0.000110 |  |
| **18** | 0.000860 |  |
| **19** | 0.000785 |  |
| **20** | 0.000247 |  |
| **21** | 0.000657 |  |
| **22** | 0.040226 |  |
| **23** | 0.054607 |  |
| **24** | 0.026151 |  |
| **25** | 0.002253 |  |
| **26** | 0.065197 |  |
| **27** | 0.057464 |  |
| **28** | 0.015938 |  |
| **29** | 0.416517 |  |
| **30** | 0.297576 |  |

| **miR-27a-3p** | | |
| --- | --- | --- |
| **NO.** | **PD** | **Ctr** |
| **1** | 0.0240881 | 0.0356305 |
| **2** | 0.1169621 | 0.018696 |
| **3** | 0.001392 | 0.2385834 |
| **4** | 0.0004897 | 3.213480 |
| **5** | 0.0001695 | 3.605134 |
| **6** | 0.0019925 | 1.738434 |
| **7** | 0.0054783 | 2.280352 |
| **8** | 0.0009733 | 0.5286601 |
| **9** | 0.0041837 | 0.198288 |
| **10** | 0.0026005 | 0.1381943 |
| **11** | 0.0171672 | 0.1335716 |
| **12** | 0.0196463 | 1.537242 |
| **13** | 0.0160015 | 0.3071998 |
| **14** | 0.0049355 | 0.0265342 |
| **15** | 0.2682813 |  |
| **16** | 0.0189116 |  |
| **17** | 0.0647786 |  |
| **18** | 0.135377 |  |
| **19** | 0.0640764 |  |
| **20** | 0.080560 |  |
| **21** | 0.0108237 |  |
| **22** | 0.0743172 |  |
| **23** | 0.0381429 |  |
| **24** | 0.0439194 |  |
| **25** | 0.0909048 |  |
| **26** | 0.0377641 |  |
| **27** | 0.0615963 |  |
| **28** | 0.021519 |  |
| **29** | 0.032378 |  |
| **30** | 0.0133819 |  |

| **miR-27b-3p** | | |
| --- | --- | --- |
| **NO.** | **PD** | **Ctr** |
| **1** | 2.822767 | 2.942210 |
| **2** | 8.918236 | 0.3178727 |
| **3** | 0.0152928 | 0.1497067 |
| **4** | 0.002690 | 1.637825 |
| **5** | 0.660226 | 2.416130 |
| **6** | 0.0050708 | 0.5341932 |
| **7** | 0.2374312 | 0.4802646 |
| **8** | 0.0004215 | 0.5351656 |
| **9** | 0.0219695 | 0.0926744 |
| **10** | 0.0074458 | 0.8996372 |
| **11** | 0.995463 | 0.1965993 |
| **12** | 2.484662 | 0.1414133 |
| **13** | 1.544347 | 3.617253 |
| **14** | 0.093757 | 0.0390548 |
| **15** | 9.444428 |  |
| **16** | 3.912410 |  |
| **17** | 6.794213 |  |
| **18** | 15.537680 |  |
| **19** | 5.110917 |  |
| **20** | 10.621040 |  |
| **21** | 2.863897 |  |
| **22** | 1.276757 |  |
| **23** | 8.785989 |  |
| **24** | 2.835587 |  |
| **25** | 7.934535 |  |
| **26** | 3.822122 |  |
| **27** | 6.619091 |  |
| **28** | 1.525621 |  |
| **29** | 2.360029 |  |
| **30** | 2.019607 |  |

1. **The values used to build graphs that related to: correlation analysis of miR-27a/b-3p levels with disease severity and duration (Fig 3).**

| **miR-27a-3p** | | | | |
| --- | --- | --- | --- | --- |
| **NO.** | **Ctr** | **HY-1** | **HY-2** | **HY-3** |
| **1** | 0.0356305 | 0.0001695 | 0.001392 | 0.024088 |
| **2** | 0.018696 | 0.0026005 | 0.000490 | 0.116962 |
| **3** | 0.2385834 | 0.0647786 | 0.001993 | 0.004184 |
| **4** | 3.213480 | 0.0640764 | 0.005478 | 0.017167 |
| **5** | 3.605134 | 0.080560 | 0.000973 | 0.019646 |
| **6** | 1.738434 | 0.0743172 | 0.016002 | 0.004936 |
| **7** | 2.280352 | 0.0381429 | 0.268281 | 0.043919 |
| **8** | 0.5286601 | 0.021519 | 0.018912 | 0.037764 |
| **9** | 0.198288 | 0.032378 | 0.010824 |  |
| **10** | 0.1381943 | 0.0133819 | 0.090905 |  |
| **11** | 0.1335716 | 0.135377 | 0.061596 |  |
| **12** | 1.537242 |  |  |  |
| **13** | 0.3071998 |  |  |  |
| **14** | 0.0265342 |  |  |  |

| **miR-27b-3p** | | | | |
| --- | --- | --- | --- | --- |
| **NO.** | **Ctr** | **HY-1** | **HY-2** | **HY-3** |
| **1** | 2.942210 | 0.660226 | 0.0152928 | 0.0036533 |
| **2** | 0.3178727 | 0.0074458 | 0.002690 | 0.2723247 |
| **3** | 0.1497067 | 6.794213 | 0.0050708 | 0.2939005 |
| **4** | 1.637825 | 5.110917 | 0.2374312 | 0.2235082 |
| **5** | 2.416130 | 10.621040 | 0.0004215 | 0.312819 |
| **6** | 0.5341932 | 1.276757 | 1.544347 | 0.4028738 |
| **7** | 0.4802646 | 8.785989 | 9.444428 | 0.0061228 |
| **8** | 0.5351656 | 1.525621 | 3.912410 | 0.0211734 |
| **9** | 0.0926744 | 2.360029 | 2.863897 |  |
| **10** | 0.8996372 | 2.019607 | 7.934535 |  |
| **11** | 0.1965993 | 15.537680 | 6.619091 |  |
| **12** | 0.1414133 |  |  |  |
| **13** | 3.617253 |  |  |  |
| **14** | 0.0390548 |  |  |  |

| **miR-27a-3p** | | | | |
| --- | --- | --- | --- | --- |
| **NO.** | **Ctr** | **0-36** | **36-60** | **>60** |
| **1** | 0.0356305 | 0.001392 | 0.0049355 | 0.0240881 |
| **2** | 0.018696 | 0.0004897 | 0.080560 | 0.1169621 |
| **3** | 0.2385834 | 0.0001695 | 0.032378 | 0.0009733 |
| **4** | 3.213480 | 0.0019925 | 0.0196463 | 0.0041837 |
| **5** | 3.605134 | 0.0054783 | 0.2682813 | 0.0026005 |
| **6** | 1.738434 | 0.0647786 |  | 0.0171672 |
| **7** | 2.280352 | 0.135377 |  | 0.0160015 |
| **8** | 0.5286601 | 0.0108237 |  | 0.0189116 |
| **9** | 0.198288 | 0.0381429 |  | 0.0640764 |
| **10** | 0.1381943 | 0.0439194 |  | 0.0743172 |
| **11** | 0.1335716 | 0.0909048 |  | 0.0377641 |
| **12** | 1.537242 | 0.021519 |  | 0.0615963 |
| **13** | 0.3071998 | 0.0133819 |  |  |
| **14** | 0.0265342 |  |  |  |

| **miR-27b-3p** | | | | |
| --- | --- | --- | --- | --- |
| **NO.** | **Ctr** | **0-36** | **36-60** | **>60** |
| **1** | 2.942210 | 0.0152928 | 2.484662 | 2.822767 |
| **2** | 0.3178727 | 0.002690 | 0.093757 | 8.918236 |
| **3** | 0.1497067 | 0.660226 | 9.444428 | 0.0004215 |
| **4** | 1.637825 | 0.0050708 | 10.621040 | 0.0219695 |
| **5** | 2.416130 | 0.2374312 | 2.360029 | 0.0074458 |
| **6** | 0.5341932 | 6.794213 |  | 0.995463 |
| **7** | 0.4802646 | 15.537680 |  | 1.544347 |
| **8** | 0.5351656 | 2.863897 |  | 3.912410 |
| **9** | 0.0926744 | 8.785989 |  | 5.110917 |
| **10** | 0.8996372 | 2.835587 |  | 1.276757 |
| **11** | 0.1965993 | 7.934535 |  | 3.822122 |
| **12** | 0.1414133 | 1.525621 |  | 6.619091 |
| **13** | 3.617253 | 2.019607 |  |  |
| **14** | 0.0390548 |  |  |  |

1. **The values used to build graphs that related to: association of miR-27a.b-3p levels with the age factor (Fig 4).**

| **miR-27a-3p** | | |
| --- | --- | --- |
| **NO.** | **Ctr** | **PD/Ctr** |
| **1** | 60.000000 | 0.0240881 |
| **2** | 62.000000 | 0.1169621 |
| **3** | 48.000000 | 0.001392 |
| **4** | 74.000000 | 0.0004897 |
| **5** | 83.000000 | 0.0001695 |
| **6** | 78.000000 | 0.0019925 |
| **7** | 73.000000 | 0.0054783 |
| **8** | 65.000000 | 0.0009733 |
| **9** | 60.000000 | 0.0041837 |
| **10** | 51.000000 | 0.0026005 |
| **11** | 51.000000 | 0.0171672 |
| **12** | 61.000000 | 0.0196463 |
| **13** | 80.000000 | 0.0160015 |
| **14** | 49.000000 | 0.0049355 |
| **15** |  | 0.2682813 |
| **16** |  | 0.0189116 |
| **17** |  | 0.0647786 |
| **18** |  | 0.135377 |
| **19** |  | 0.0640764 |
| **20** |  | 0.080560 |
| **21** |  | 0.0108237 |
| **22** |  | 0.0743172 |
| **23** |  | 0.0381429 |
| **24** |  | 0.0439194 |
| **25** |  | 0.0909048 |
| **26** |  | 0.0377641 |
| **27** |  | 0.0615963 |
| **28** |  | 0.021519 |
| **29** |  | 0.032378 |
| **30** |  | 0.0133819 |
| **31** |  | 0.0356305 |
| **32** |  | 0.018696 |
| **33** |  | 0.2385834 |
| **34** |  | 3.213480 |
| **35** |  | 3.605134 |
| **36** |  | 1.738434 |
| **37** |  | 2.280352 |
| **38** |  | 0.5286601 |
| **39** |  | 0.198288 |
| **40** |  | 0.1381943 |
| **41** |  | 0.1335716 |
| **42** |  | 1.537242 |
| **43** |  | 0.3071998 |
| **44** |  | 0.0265342 |

| **miR-27b-3p** | | |
| --- | --- | --- |
| **NO.** | **Ctr** | **PD/Ctr** |
| **1** | 60.000000 | 2.822767 |
| **2** | 62.000000 | 8.918236 |
| **3** | 48.000000 | 0.0152928 |
| **4** | 74.000000 | 0.002690 |
| **5** | 83.000000 | 0.660226 |
| **6** | 78.000000 | 0.0050708 |
| **7** | 73.000000 | 0.2374312 |
| **8** | 65.000000 | 0.0004215 |
| **9** | 60.000000 | 0.0219695 |
| **10** | 51.000000 | 0.0074458 |
| **11** | 51.000000 | 0.995463 |
| **12** | 61.000000 | 2.484662 |
| **13** | 80.000000 | 1.544347 |
| **14** | 49.000000 | 0.093757 |
| **15** |  | 9.444428 |
| **16** |  | 3.912410 |
| **17** |  | 6.794213 |
| **18** |  | 15.537680 |
| **19** |  | 5.110917 |
| **20** |  | 10.621040 |
| **21** |  | 2.863897 |
| **22** |  | 1.276757 |
| **23** |  | 8.785989 |
| **24** |  | 2.835587 |
| **25** |  | 7.934535 |
| **26** |  | 3.822122 |
| **27** |  | 6.619091 |
| **28** |  | 1.525621 |
| **29** |  | 2.360029 |
| **30** |  | 2.019607 |
| **31** |  | 2.942210 |
| **32** |  | 0.3178727 |
| **33** |  | 0.1497067 |
| **34** |  | 1.637825 |
| **35** |  | 2.416130 |
| **36** |  | 0.5341932 |
| **37** |  | 0.4802646 |
| **38** |  | 0.5351656 |
| **39** |  | 0.0926744 |
| **40** |  | 0.8996372 |
| **41** |  | 0.1965993 |
| **42** |  | 0.1414133 |
| **43** |  | 3.617253 |
| **44** |  | 0.0390548 |

| **miR-27a-3p** | | |
| --- | --- | --- |
| **NO.** | **PD** | **PD/Ctr** |
| **1** | 39.000000 | 0.0240881 |
| **2** | 65.000000 | 0.1169621 |
| **3** | 62.000000 | 0.001392 |
| **4** | 49.000000 | 0.0004897 |
| **5** | 69.000000 | 0.0001695 |
| **6** | 41.000000 | 0.0019925 |
| **7** | 78.000000 | 0.0054783 |
| **8** | 57.000000 | 0.0009733 |
| **9** | 60.000000 | 0.0041837 |
| **10** | 60.000000 | 0.0026005 |
| **11** | 73.000000 | 0.0171672 |
| **12** | 70.000000 | 0.0196463 |
| **13** | 78.000000 | 0.0160015 |
| **14** | 78.000000 | 0.0049355 |
| **15** | 70.000000 | 0.2682813 |
| **16** | 40.000000 | 0.0189116 |
| **17** | 72.000000 | 0.0647786 |
| **18** | 64.000000 | 0.135377 |
| **19** | 51.000000 | 0.0640764 |
| **20** | 62.000000 | 0.080560 |
| **21** | 75.000000 | 0.0108237 |
| **22** | 57.000000 | 0.0743172 |
| **23** | 60.000000 | 0.0381429 |
| **24** | 42.000000 | 0.0439194 |
| **25** | 51.000000 | 0.0909048 |
| **26** | 75.000000 | 0.0377641 |
| **27** | 67.000000 | 0.0615963 |
| **28** | 63.000000 | 0.021519 |
| **29** | 62.000000 | 0.032378 |
| **30** | 57.000000 | 0.0133819 |
| **31** |  | 0.0356305 |
| **32** |  | 0.018696 |
| **33** |  | 0.2385834 |
| **34** |  | 3.213480 |
| **35** |  | 3.605134 |
| **36** |  | 1.738434 |
| **37** |  | 2.280352 |
| **38** |  | 0.5286601 |
| **39** |  | 0.198288 |
| **40** |  | 0.1381943 |
| **41** |  | 0.1335716 |
| **42** |  | 1.537242 |
| **43** |  | 0.3071998 |
| **44** |  | 0.0265342 |

| **miR-27b-3p** | | |
| --- | --- | --- |
| **NO.** | **PD** | **PD/Ctr** |
| **1** | 39.000000 | 2.822767 |
| **2** | 65.000000 | 8.918236 |
| **3** | 62.000000 | 0.0152928 |
| **4** | 49.000000 | 0.002690 |
| **5** | 69.000000 | 0.660226 |
| **6** | 41.000000 | 0.0050708 |
| **7** | 78.000000 | 0.2374312 |
| **8** | 57.000000 | 0.0004215 |
| **9** | 60.000000 | 0.0219695 |
| **10** | 60.000000 | 0.0074458 |
| **11** | 73.000000 | 0.995463 |
| **12** | 70.000000 | 2.484662 |
| **13** | 78.000000 | 1.544347 |
| **14** | 78.000000 | 0.093757 |
| **15** | 70.000000 | 9.444428 |
| **16** | 40.000000 | 3.912410 |
| **17** | 72.000000 | 6.794213 |
| **18** | 64.000000 | 15.537680 |
| **19** | 51.000000 | 5.110917 |
| **20** | 62.000000 | 10.621040 |
| **21** | 75.000000 | 2.863897 |
| **22** | 57.000000 | 1.276757 |
| **23** | 60.000000 | 8.785989 |
| **24** | 42.000000 | 2.835587 |
| **25** | 51.000000 | 7.934535 |
| **26** | 75.000000 | 3.822122 |
| **27** | 67.000000 | 6.619091 |
| **28** | 63.000000 | 1.525621 |
| **29** | 62.000000 | 2.360029 |
| **30** | 57.000000 | 2.019607 |
| **31** |  | 2.942210 |
| **32** |  | 0.3178727 |
| **33** |  | 0.1497067 |
| **34** |  | 1.637825 |
| **35** |  | 2.416130 |
| **36** |  | 0.5341932 |
| **37** |  | 0.4802646 |
| **38** |  | 0.5351656 |
| **39** |  | 0.0926744 |
| **40** |  | 0.8996372 |
| **41** |  | 0.1965993 |
| **42** |  | 0.1414133 |
| **43** |  | 3.617253 |
| **44** |  | 0.0390548 |

1. **The values used to build graphs that related to: correlation between *SRRM2* and miR-27a/b-3p levels, and their ROC curve (Fig 5).**

| **Correlation (PD)** | | |
| --- | --- | --- |
| **NO.** | **miR-27b-3p** | **SRRM2** |
| **1** | 2.822767 | 0.008764 |
| **2** | 8.918236 | 0.575051 |
| **3** | 0.0152928 | 0.307795 |
| **4** | 0.002690 | 0.003636 |
| **5** | 0.660226 | 0.077985 |
| **6** | 0.0050708 | 0.292859 |
| **7** | 0.2374312 | 0.545028 |
| **8** | 0.0004215 | 1.002872 |
| **9** | 0.0219695 | 0.754366 |
| **10** | 0.0074458 | 0.098385 |
| **11** | 0.995463 | 0.440114 |
| **12** | 2.484662 | 0.486236 |
| **13** | 1.544347 | 1.378699 |
| **14** | 0.093757 | 0.793084 |
| **15** | 9.444428 | 0.609729 |
| **16** | 3.912410 | 0.001933 |
| **17** | 6.794213 | 0.000110 |
| **18** | 15.537680 | 0.000860 |
| **19** | 5.110917 | 0.000785 |
| **20** | 10.621040 | 0.000247 |
| **21** | 2.863897 | 0.000657 |
| **22** | 1.276757 | 0.040226 |
| **23** | 8.785989 | 0.054607 |
| **24** | 2.835587 | 0.026151 |
| **25** | 7.934535 | 0.002253 |
| **26** | 3.822122 | 0.065197 |
| **27** | 6.619091 | 0.057464 |
| **28** | 1.525621 | 0.015938 |
| **29** | 2.360029 | 0.416517 |
| **30** | 2.019607 | 0.297576 |

| **Correlation (PD)** | | |
| --- | --- | --- |
| **NO.** | **miR-27a-3p** | **SRRM2** |
| **1** | 0.0240881 | 0.008764 |
| **2** | 0.1169621 | 0.575051 |
| **3** | 0.001392 | 0.307795 |
| **4** | 0.0004897 | 0.003636 |
| **5** | 0.0001695 | 0.077985 |
| **6** | 0.0019925 | 0.292859 |
| **7** | 0.0054783 | 0.545028 |
| **8** | 0.0009733 | 1.002872 |
| **9** | 0.0041837 | 0.754366 |
| **10** | 0.0026005 | 0.098385 |
| **11** | 0.0171672 | 0.440114 |
| **12** | 0.0196463 | 0.486236 |
| **13** | 0.0160015 | 1.378699 |
| **14** | 0.0049355 | 0.793084 |
| **15** | 0.2682813 | 0.609729 |
| **16** | 0.0189116 | 0.001933 |
| **17** | 0.0647786 | 0.000110 |
| **18** | 0.135377 | 0.000860 |
| **19** | 0.0640764 | 0.000785 |
| **20** | 0.080560 | 0.000247 |
| **21** | 0.0108237 | 0.000657 |
| **22** | 0.0743172 | 0.040226 |
| **23** | 0.0381429 | 0.054607 |
| **24** | 0.0439194 | 0.026151 |
| **25** | 0.0909048 | 0.002253 |
| **26** | 0.0377641 | 0.065197 |
| **27** | 0.0615963 | 0.057464 |
| **28** | 0.021519 | 0.015938 |
| **29** | 0.032378 | 0.416517 |
| **30** | 0.0133819 | 0.297576 |

| **Correlation (PD)** | | |
| --- | --- | --- |
| **NO.** | **miR-27a-3p** | **miR-27b-3p** |
| **1** | 0.0240881 | 2.822767 |
| **2** | 0.1169621 | 8.918236 |
| **3** | 0.001392 | 0.0152928 |
| **4** | 0.0004897 | 0.002690 |
| **5** | 0.0001695 | 0.660226 |
| **6** | 0.0019925 | 0.0050708 |
| **7** | 0.0054783 | 0.2374312 |
| **8** | 0.0009733 | 0.0004215 |
| **9** | 0.0041837 | 0.0219695 |
| **10** | 0.0026005 | 0.0074458 |
| **11** | 0.0171672 | 0.995463 |
| **12** | 0.0196463 | 2.484662 |
| **13** | 0.0160015 | 1.544347 |
| **14** | 0.0049355 | 0.093757 |
| **15** | 0.2682813 | 9.444428 |
| **16** | 0.0189116 | 3.912410 |
| **17** | 0.0647786 | 6.794213 |
| **18** | 0.135377 | 15.537680 |
| **19** | 0.0640764 | 5.110917 |
| **20** | 0.080560 | 10.621040 |
| **21** | 0.0108237 | 2.863897 |
| **22** | 0.0743172 | 1.276757 |
| **23** | 0.0381429 | 8.785989 |
| **24** | 0.0439194 | 2.835587 |
| **25** | 0.0909048 | 7.934535 |
| **26** | 0.0377641 | 3.822122 |
| **27** | 0.0615963 | 6.619091 |
| **28** | 0.021519 | 1.525621 |
| **29** | 0.032378 | 2.360029 |
| **30** | 0.0133819 | 2.019607 |

| **Correlation (PD/Ctr)** | | |
| --- | --- | --- |
| **NO.** | **miR-27b-3p** | **SRRM2** |
| **1** | 2.822767 | 0.008764 |
| **2** | 8.918236 | 0.575051 |
| **3** | 0.0152928 | 0.307795 |
| **4** | 0.002690 | 0.003636 |
| **5** | 0.660226 | 0.077985 |
| **6** | 0.0050708 | 0.292859 |
| **7** | 0.2374312 | 0.545028 |
| **8** | 0.0004215 | 1.002872 |
| **9** | 0.0219695 | 0.754366 |
| **10** | 0.0074458 | 0.098385 |
| **11** | 0.995463 | 0.440114 |
| **12** | 2.484662 | 0.486236 |
| **13** | 1.544347 | 1.378699 |
| **14** | 0.093757 | 0.793084 |
| **15** | 9.444428 | 0.609729 |
| **16** | 3.912410 | 0.001933 |
| **17** | 6.794213 | 0.000110 |
| **18** | 15.537680 | 0.000860 |
| **19** | 5.110917 | 0.000785 |
| **20** | 10.621040 | 0.000247 |
| **21** | 2.863897 | 0.000657 |
| **22** | 1.276757 | 0.040226 |
| **23** | 8.785989 | 0.054607 |
| **24** | 2.835587 | 0.026151 |
| **25** | 7.934535 | 0.002253 |
| **26** | 3.822122 | 0.065197 |
| **27** | 6.619091 | 0.057464 |
| **28** | 1.525621 | 0.015938 |
| **29** | 2.360029 | 0.416517 |
| **30** | 2.019607 | 0.297576 |
| **31** | 2.942210 | 2.107179 |
| **32** | 0.3178727 | 2.418022 |
| **33** | 0.1497067 | 0.318271 |
| **34** | 1.637825 | 0.017666 |
| **35** | 2.416130 | 0.891364 |
| **36** | 0.5341932 | 0.540961 |
| **37** | 0.4802646 | 0.058729 |
| **38** | 0.5351656 | 0.779865 |
| **39** | 0.0926744 | 1.722561 |
| **40** | 0.8996372 | 1.132863 |
| **41** | 0.1965993 | 0.685435 |
| **42** | 0.1414133 | 0.365083 |
| **43** | 3.617253 | 0.492398 |
| **44** | 0.0390548 | 2.469601 |

| **Correlation (Ctr)** | | |
| --- | --- | --- |
| **NO.** | **miR-27a-3p** | **SRRM2** |
| **1** | 0.0356305 | 2.107179 |
| **2** | 0.018696 | 2.418022 |
| **3** | 0.2385834 | 0.318271 |
| **4** | 3.213480 | 0.017666 |
| **5** | 3.605134 | 0.891364 |
| **6** | 1.738434 | 0.540961 |
| **7** | 2.280352 | 0.058729 |
| **8** | 0.5286601 | 0.779865 |
| **9** | 0.198288 | 1.722561 |
| **10** | 0.1381943 | 1.132863 |
| **11** | 0.1335716 | 0.685435 |
| **12** | 1.537242 | 0.365083 |
| **13** | 0.3071998 | 0.492398 |
| **14** | 0.0265342 | 2.469601 |

| **ROC curve of SRRM2** | | |
| --- | --- | --- |
| **NO.** | **Ctr** | **PD** |
| 1 | 2.107179 | 0.008764 |
| **2** | 2.418022 | 0.575051 |
| **3** | 0.318271 | 0.307795 |
| **4** | 0.017666 | 0.003636 |
| **5** | 0.891364 | 0.077985 |
| **6** | 0.540961 | 0.292859 |
| **7** | 0.058729 | 0.545028 |
| **8** | 0.779865 | 1.002872 |
| **9** | 1.722561 | 0.754366 |
| **10** | 1.132863 | 0.098385 |
| **11** | 0.685435 | 0.440114 |
| **12** | 0.365083 | 0.486236 |
| **13** | 0.492398 | 1.378699 |
| **14** | 2.469601 | 0.793084 |
| **15** |  | 0.609729 |
| **16** |  | 0.001933 |
| **17** |  | 0.000110 |
| **18** |  | 0.000860 |
| **19** |  | 0.000785 |
| **20** |  | 0.000247 |
| **21** |  | 0.000657 |
| **22** |  | 0.040226 |
| **23** |  | 0.054607 |
| **24** |  | 0.026151 |
| **25** |  | 0.002253 |
| **26** |  | 0.065197 |
| **27** |  | 0.057464 |
| **28** |  | 0.015938 |
| **29** |  | 0.416517 |
| **30** |  | 0.297576 |

| **ROC curve of miR-27b-3p** | | |
| --- | --- | --- |
| **NO.** | **Ctr** | **PD** |
| 1 | 2.942210 | 2.822767 |
| **2** | 0.3178727 | 8.918236 |
| **3** | 0.1497067 | 0.0152928 |
| **4** | 1.637825 | 0.002690 |
| **5** | 2.416130 | 0.660226 |
| **6** | 0.5341932 | 0.0050708 |
| **7** | 0.4802646 | 0.2374312 |
| **8** | 0.5351656 | 0.0004215 |
| **9** | 0.0926744 | 0.0219695 |
| **10** | 0.8996372 | 0.0074458 |
| **11** | 0.1965993 | 0.995463 |
| **12** | 0.1414133 | 2.484662 |
| **13** | 3.617253 | 1.544347 |
| **14** | 0.0390548 | 0.093757 |
| **15** |  | 9.444428 |
| **16** |  | 3.912410 |
| **17** |  | 6.794213 |
| **18** |  | 15.537680 |
| **19** |  | 5.110917 |
| **20** |  | 10.621040 |
| **21** |  | 2.863897 |
| **22** |  | 1.276757 |
| **23** |  | 8.785989 |
| **24** |  | 2.835587 |
| **25** |  | 7.934535 |
| **26** |  | 3.822122 |
| **27** |  | 6.619091 |
| **28** |  | 1.525621 |
| **29** |  | 2.360029 |
| **30** |  | 2.019607 |

| **ROC curve of miR-27a-3p** | | |
| --- | --- | --- |
| **NO.** | **Ctr** | **PD** |
| 1 | 0.0356305 | 0.0240881 |
| **2** | 0.018696 | 0.1169621 |
| **3** | 0.2385834 | 0.001392 |
| **4** | 3.213480 | 0.0004897 |
| **5** | 3.605134 | 0.0001695 |
| **6** | 1.738434 | 0.0019925 |
| **7** | 2.280352 | 0.0054783 |
| **8** | 0.5286601 | 0.0009733 |
| **9** | 0.198288 | 0.0041837 |
| **10** | 0.1381943 | 0.0026005 |
| **11** | 0.1335716 | 0.0171672 |
| **12** | 1.537242 | 0.0196463 |
| **13** | 0.3071998 | 0.0160015 |
| **14** | 0.0265342 | 0.0049355 |
| **15** |  | 0.2682813 |
| **16** |  | 0.0189116 |
| **17** |  | 0.0647786 |
| **18** |  | 0.135377 |
| **19** |  | 0.0640764 |
| **20** |  | 0.080560 |
| **21** |  | 0.0108237 |
| **22** |  | 0.0743172 |
| **23** |  | 0.0381429 |
| **24** |  | 0.0439194 |
| **25** |  | 0.0909048 |
| **26** |  | 0.0377641 |
| **27** |  | 0.0615963 |
| **28** |  | 0.021519 |
| **29** |  | 0.032378 |
| **30** |  | 0.0133819 |

| **ROC curve of miR-27a- 3p/miR-27b-3p** | | |
| --- | --- | --- |
| **NO.** | **miR-27a-3p** | **miR-27b-3p** |
| **1** | 0.0240881 | 2.822767 |
| **2** | 0.1169621 | 8.918236 |
| **3** | 0.001392 | 0.0152928 |
| **4** | 0.0004897 | 0.002690 |
| **5** | 0.0001695 | 0.660226 |
| **6** | 0.0019925 | 0.0050708 |
| **7** | 0.0054783 | 0.2374312 |
| **8** | 0.0009733 | 0.0004215 |
| **9** | 0.0041837 | 0.0219695 |
| **10** | 0.0026005 | 0.0074458 |
| **11** | 0.0171672 | 0.995463 |
| **12** | 0.0196463 | 2.484662 |
| **13** | 0.0160015 | 1.544347 |
| **14** | 0.0049355 | 0.093757 |
| **15** | 0.2682813 | 9.444428 |
| **16** | 0.0189116 | 3.912410 |
| **17** | 0.0647786 | 6.794213 |
| **18** | 0.135377 | 15.537680 |
| **19** | 0.0640764 | 5.110917 |
| **20** | 0.080560 | 10.621040 |
| **21** | 0.0108237 | 2.863897 |
| **22** | 0.0743172 | 1.276757 |
| **23** | 0.0381429 | 8.785989 |
| **24** | 0.0439194 | 2.835587 |
| **25** | 0.0909048 | 7.934535 |
| **26** | 0.0377641 | 3.822122 |
| **27** | 0.0615963 | 6.619091 |
| **28** | 0.021519 | 1.525621 |
| **29** | 0.032378 | 2.360029 |
| **30** | 0.0133819 | 2.019607 |
| **31** | 0.0356305 | 2.942210 |
| **32** | 0.018696 | 0.3178727 |
| **33** | 0.2385834 | 0.1497067 |
| **34** | 3.213480 | 1.637825 |
| **35** | 3.605134 | 2.416130 |
| **36** | 1.738434 | 0.5341932 |
| **37** | 2.280352 | 0.4802646 |
| **38** | 0.5286601 | 0.5351656 |
| **39** | 0.198288 | 0.0926744 |
| **40** | 0.1381943 | 0.8996372 |
| **41** | 0.1335716 | 0.1965993 |
| **42** | 1.537242 | 0.1414133 |
| **43** | 0.3071998 | 3.617253 |
| **44** | 0.0265342 | 0.0390548 |
